# Supplementary material for: The Release of Inflammatory Mediators from Acid-Stimulated Mesenchymal Stromal Cells Favours Tumour Invasiveness and Metastasis in Osteosarcoma
Source: Cancers (Basel). 2021 Nov 22;13(22):5855. doi: 10.3390/cancers13225855 (PMC8616358; doi:10.3390/cancers13225855)
Supplement: Supplementary file 1 [file cancers-13-05855-s001.zip › cancers-1447154-supplementary.pdf]

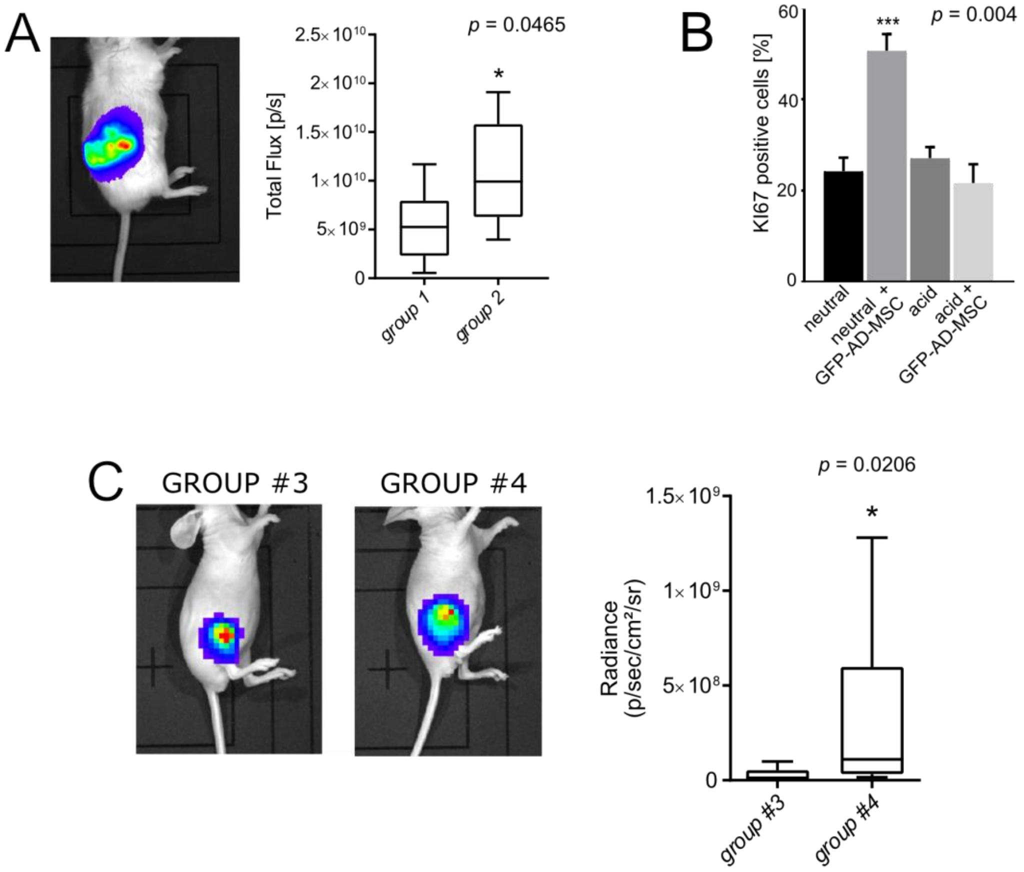

Figure S1. GFP-AD-MSC enhance tumour growth with an acid-independent mechanism. (a) Left panel: representative image of luciferase assay in a subcutaneously injected OS xenograft after 15 days; right panel: tumour growth in group #1 and #2 of the animal trial n. 2 (see Table 2), as measured by the Total flux (n = 6, $^{*}p < 0.05$, Mann Whitney U test, median and percentiles are shown); **(b)** n. of Ki67 positive cells that were also negative for GFP, in luc-143b spheroids, enriched or not with GFP-AD-MSC, and cultured in medium at different pH (n = 6, $^{***}p < 0.001$, Mann Whitney U test, mean ± SEM); **(c)** Left panel: representative images of luciferase assay in a intriatibially injected OS xenografts after 15 days; right panel: tumour growth in the tibia in the group #3 and #4 of the animal trial n. 6 (see Table 2) at 8 days, as measured by the Total flux (n = 6, $^{*}p < 0.05$, Mann Whitney U test, median and percentiles are shown).

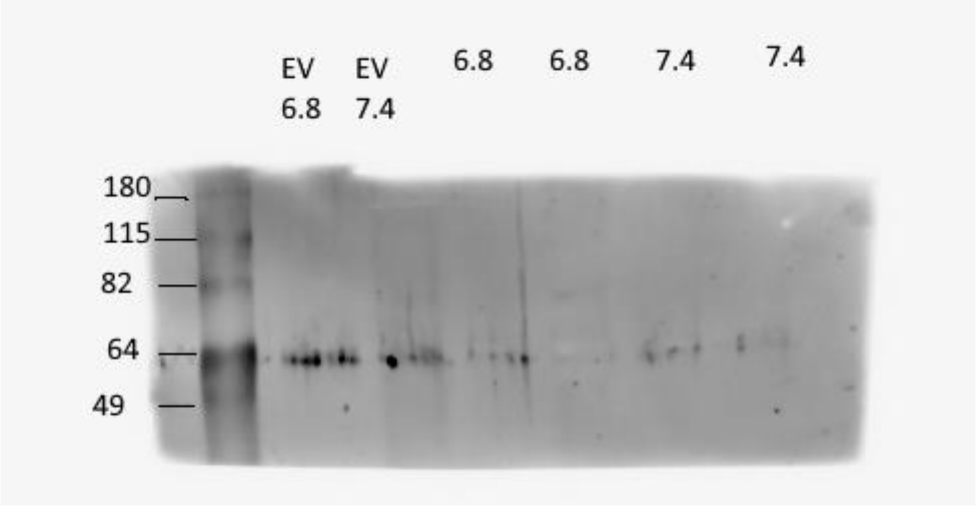

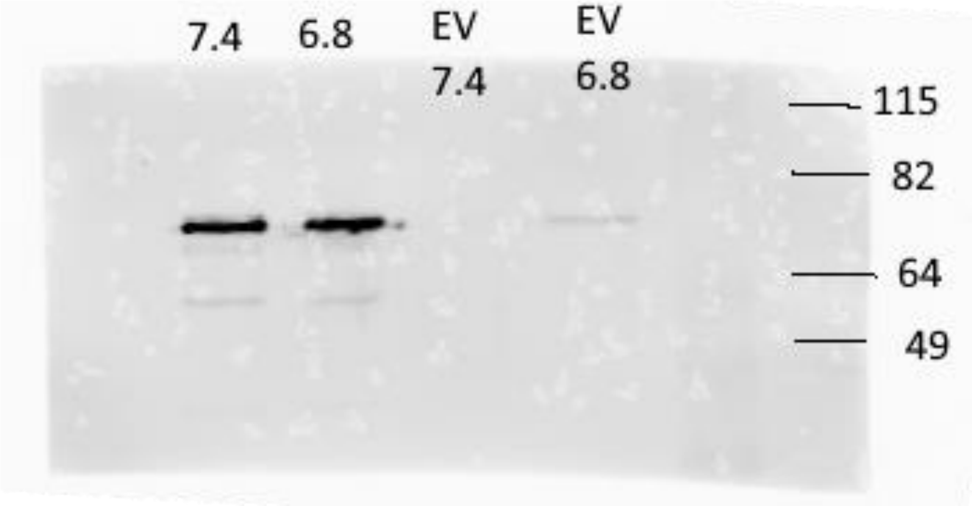

Figure S2: Original Images of Western Blot analysis of Fig. 1G.

Table S1. List of primers.

Definition	Locus	Reference GeneBank	Forward	Reverse	Aplicon Length
Human 18S ribosomal	X03205	X03205.1|X03205	5′-gcaattattccccatgaacg-3′	5′-gggacttaatcaacgcaagc-3′	68 nt
Homo sapiens glyceraldehyde-3-phosphate dehydrogenase (GAPDH)	NM_002046	NM_002046.3	5′-agccacatcgctcagacac-3′	5′-gcccaatacgaccaaatcc-3′	66 nt
Homo sapiens tyrosine 3-monooxygenase/tryptophan 5-monooxygenase activation protein zeta (YWHAZ), transcript variant 1	NM_003406	NM_003406.3	5′-ccgttacttggctgaggttg-3′	5′-tgcttgttgtgactgatcgac-3′	67 nt
Human beta-glucuronidase (GUSB)	HUMGLCB	M15182.1|M15182	5′-cgccctgcctatctgtattc-3′	5′-tccccacagggagtgtgtag-3′	91 nt
Homo sapiens ATPase H+ transporting V1 subunit B2 (ATP6V1B2)	NM_001693	NM_001693.3	5′-tggccgaagacttccttg-3′	5′-ccgaaatgccagtctgaatc-3′	90 nt
Homo sapiens interleukin 6 (IL6)	NM_000600	NM_000600.3	5′-gatgagtacaaaagtcctgatcca-3′	5′-ctgcagccactggttctgt-3′	130 nt
